# Aging Gut-Brain Interactions: Pro-Inflammatory Gut Bacteria Are Elevated in Fecal Samples from Individuals Living with Alzheimer’s Dementia

**DOI:** 10.3390/geriatrics10020037

**Published:** 2025-03-07

**Authors:** Alison I. C. Donaldson, Claire L. Fyfe, Jennifer C. Martin, Ellen E. Smith, Graham W. Horgan, Phyo K. Myint, Alexandra M. Johnstone, Karen P. Scott

**Affiliations:** 1 Institute of Applied Health Sciences, School of Medicine, Medical Sciences and Nutrition, University of Aberdeen, Aberdeen AB25 2ZD, UK; phyo.myint@abdn.ac.uk; 2 The Rowett Institute, School of Medicine, Medical Sciences and Nutrition, University of Aberdeen, Aberdeen AB25 2ZD, UK; alex.johnstone@abdn.ac.uk (A.M.J.); k.scott@abdn.ac.uk (K.P.S.); 3 Centre for Genome Enabled Biology and Medicine, University of Aberdeen, 23 St Machar Drive, Aberdeen AB24 3FX, UK; 4 Biomathematics and Statistics Scotland (BioSS), Aberdeen AB25 2ZD, UK

**Keywords:** brain, Alzheimer’s disease, gut-brain axis, fecal microbiota

## Abstract

**Background/Objectives**: Alzheimer’s disease (AD) is the most common form of dementia, characterized by an irreversible decline in cognitive function. The pathogenesis of several neurodegenerative disorders has been linked to changes in the gut microbiota, transmitted through the gut-brain axis. **Methods**: We set out to establish by case-control study methodology whether there were any differences in the composition and/or function of the gut microbiota between older resident adults in care homes with or without an AD diagnosis via analysis of the microbial composition from fecal samples. **Results**: The microbial composition, determined by 16S rRNA gene profiling, indicated that AD sufferers had significantly increased proportions of *Escherichia/Shigella* and *Clostridium_sensu_stricto_1*, and significantly decreased proportions of *Bacteroides*, *Faecalibacterium*, *Blautia*, and *Roseburia* species. The increase in potentially pro-inflammatory bacteria was consistent with slightly higher concentrations of calprotectin, a biomarker of gut inflammation. Fecal concentrations of most microbial metabolites measured were similar across groups, although participants with AD had significantly increased proportions of the branched-chain fatty acid, iso-butyrate, and lower overall concentrations of total short chain fatty acids. **Conclusions**: Participants with Alzheimer’s disease have several key differences within their gut microbiota profile, in contrast to care home residents without Alzheimer’s disease. The altered microbiome included both compositional and functional changes linked to poorer health and gut inflammation.

## 1. Introduction

The aging global population is facing a growing burden of age-related diseases, including dementia, with an estimated worldwide prevalence of over 55 million dementia cases, projected to rise to 78 million cases by 2030 [1,2,3]. While Alzheimer’s disease (AD) is the leading cause of dementia, accounting for 50–75% of all dementia cases, over half of individuals with AD are likely to have brain changes of another dementia type at time of diagnosis termed ‘mixed dementia’ [1,4]. In clinical practice, the most appropriate diagnosis of dementia type is made based on symptoms and clinical history, and mixed dementia becomes more prevalent with advancing age due to the accumulation of cerebrovascular disease [4,5].

AD is characterized by the accumulation of abnormal proteins (including β-amyloid peptides) and progressive brain atrophy [6,7]. These characteristic brain changes are thought to start twenty years or more before symptoms are noticed [4,6]. The key clinical symptoms initially include memory impairment, apathy, and depression, before progressing to impaired communication, executive function, and behavior, before eventually affecting mobility, speech, and swallowing in the latter stages [4,8,9]. Over the course of AD, the vast majority of people (50–90%) will experience some form of behavioral or psychological symptoms of dementia (BPSD) [10,11], including sleep disturbance, agitation, aggression, repetitive calling out, and apathy [12]. These challenging behaviors increase the likelihood of a person requiring institutional care [13].

There are multiple contributing factors thought to lead to the development of AD, including increasing age, genetics, and family history. However, there are also several associated modifiable risk factors which may prevent or delay up to 45% of cases, these include exercise, vision and hearing loss, cholesterol levels, diabetes, obesity, depression, smoking, education, blood pressure, being socially and mentally active, and diet [14,15].

Despite the global prevalence of AD, widely available drug treatments mainly target symptoms by increasing neurotransmitters in the brain (e.g., Rivastigmine, Donepezil) [16,17]. There has been recent progress with monoclonal antibody-based treatments which target amyloid plaques in the brains of those with AD (Lecanemab, Donanemab), but while the MHRA has recently approved use of Lecanemab and Donanemab in the UK for those in the early stages of AD and who have one or no copies of the apolipoprotein E4 gene, access is currently limited to private prescriptions [18,19]. Therefore, any novel therapeutic or preventative strategies, including diet and lifestyle, that can delay the onset and progression of AD and contribute to a healthy life expectancy will help to ease the global burden of an aging population.

Emerging research suggests a relationship between cognitive aging, neurodegenerative diseases, and the gut microbiota [20,21,22,23,24]. This was clearly illustrated through recent transplantation experiments. When the fecal microbiota from participants with AD, or age-matched healthy controls, was transplanted into young adult rats, those receiving the AD microbiota exhibited behavioral changes affecting memory functions and mood [25]. Although there is currently little evidence for the role of diet in management of AD, specific diets have been shown to reduce the incidence of AD in the older adult population [26]. As we age, our diet typically changes which, along with the effects of aging on the physiology and functioning of the gastrointestinal (GI) tract, may impact the composition and activity of the gut microbiota [27].

**Figure 1:**
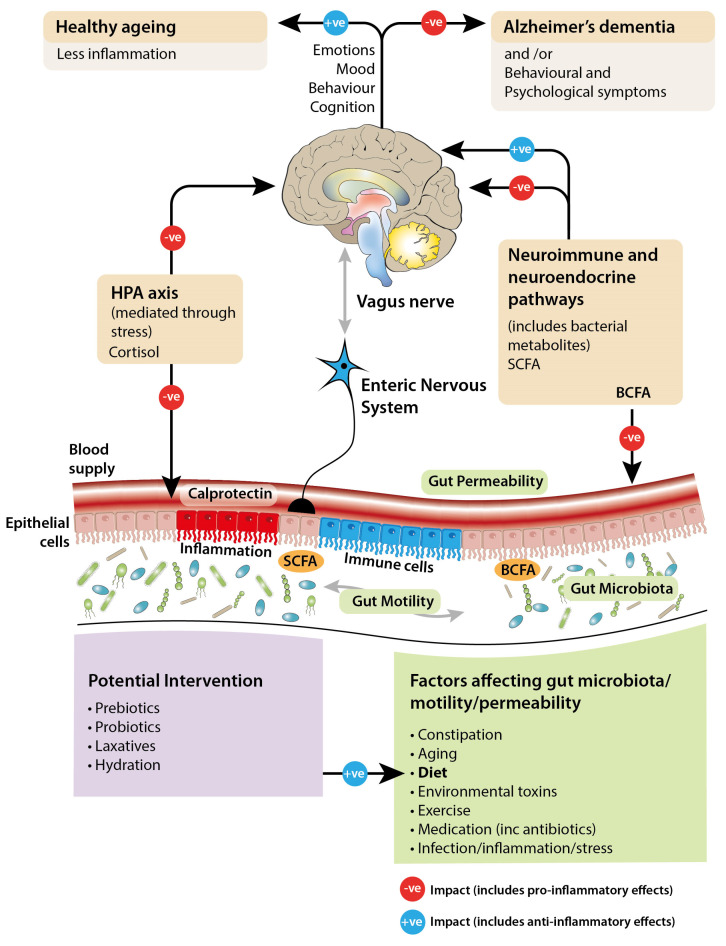
Diagram illustrating the hypothesized bidirectional signaling between the gut and the brain, and the potential factors affecting this.

Among community-dwelling older adults, a correlation between altered gut microbiota and frailty rather than chronological age has been found, with changes in dietary composition and diversity being considered the main drivers of the shifts in gut bacterial profile [28,29]. The onset and progression of age-associated inflammation can be influenced by changes in the equilibrium of the gut microbiota either by increasing production of pro-inflammatory mediators (see Figure 1) or lowering production of those that are anti-inflammatory [30,31]. Studies comparing the gut microbial profiles of frail and healthy older people show that bacterial species associated with inflammation are more prevalent in the former group and are also increased in prevalence in centenarians [20,32].

Research focused specifically on AD has shown that increased levels of neurotoxic proteins in the brain and circulating pro-inflammatory cytokines correlate with a lower abundance of the butyrate-producing anti-inflammatory bacterium *Eubacterium rectale* and higher levels of pro-inflammatory *Escherichia coli* and *Shigella* [33]. This is a key finding as neuro-inflammation is associated with cognitive decline, but research is needed to understand any associations between cognition and the gut microbiota composition [34].

At present, it is unclear if modifying the gut microbiota profile, and thus metabolic products, through dietary changes can help prevent or slow down the progression of AD. Although there have now been many studies investigating alterations in the gut microbiota associated with AD, few have focused on different dementia subtypes and examined the gut microbiota profile in those diagnosed with AD and experiencing challenging behaviors [11]. With increasing evidence that the gut microbiota may mediate the interaction between nutrition and brain function, and that the composition of the microbiota correlates with diet and health in older people, our research question in the *Aging Gut-Brain* proof-of-concept case-control study (AGE-GB) tested the hypothesis that differences exist in the gut microbiota composition and function between healthy aging participants and those with dementia, with an additional focus on comparing profiles of individuals displaying challenging behaviors.

## 2. Materials and Methods

### 2.1. Study Design

Ethical approval was obtained for the Aging Gut Brain study from the Scotland A Research Ethics Committee which considered applications concerning vulnerable groups (in this case, participants who could not consent by themselves). The study was registered with the online clinical trials database with identifier code NCT03593941 (URL: www.clinicaltrials.gov (accessed on 31 January 2020). Following statistical power calculations detecting a 30% difference in iso-butyrate, we aimed to recruit 20 participants of similar ages from three groups of care home residents: healthy controls (no dementia); those with AD; those with AD and BPSD (for the purposes of this study shall be referred to as ADCB, representing AD with Challenging Behavior). Forty-six care homes in Aberdeen and Aberdeenshire were contacted to take part in the study but only sixteen were willing to participate. Care home staff were asked to identify potentially suitable participants and National Health Service (NHS) research nurses facilitated their recruitment and consent, with simplified participant information provided to support the inclusion of participants in the process where possible, and consent obtained by proxy for those lacking capacity. Whenever a diagnosis of AD was not clear, the participant’s general practitioner was asked to provide confirmation (Further details available in: Supplementary Methods: participant selection and enrolment). Each participant was asked to provide two fecal samples at least a week apart, as described in the literature previously [35]. Unfortunately, some subjects recruited did not provide any samples while others, particularly in the AD groups, did not provide second replicate samples. The final numbers of participants providing at least one sample were: C (*n* = 19/20), AD (*n* = 14/20), ADCB (*n* = 10/20) (Table 1). The low number of participants providing samples in the AD groups meant that we performed primary analysis comparing controls (*n* = 19) against combined AD groups (*n* = 24).

### 2.2. Fecal Sample Collection

Fecal sample collection kits were provided containing sample pots (Fecontainer, AT Medical BV, Enschede, The Netherlands) which sit on a toilet/commode, plastic liners, gloves, labels, and fully standardized instructions. After collection (with assistance from care home staff), the pot was sealed and the kit collected from the care home by a member of the research team and processed in the laboratory within 12 h. Fresh faeces (5 g) were diluted 1:2 (*w*/*v*) with phosphate-buffered saline (1 × PBS, pH 7.4, containing 30% glycerol) and homogenised by gentleMACS Dissociator (Miltenyi Biotec, Bergisch Gladbach, Germany). Secondly, 3 mL aliquots were stored and frozen at −20 °C for subsequent short-chain fatty acid quantification to assess the activity of the gut microbiota. One 450 µL aliquot was used immediately for DNA extraction followed by 16S rRNA gene sequencing to determine the microbiota composition down to genus level, while further backup 600 µL aliquots were stored at −70 °C.

### 2.3. Calprotectin Concentrations

Concentrations of the inflammatory biomarker, calprotectin, were estimated using a calprotectin ELISA kit following the manufacturer’s instructions (CALPROLAB Calprotectin ELISA ALP, REF: CALPO218, Firefly Scientific, Manchester, UK). Absorbances of duplicate assays were measured using a Tecan Safire 2 spectrophotometer, reading at 405 nm. Actual concentrations were calculated based on standard curve absorbances.

### 2.4. Short-Chain and Branched-Chain Fatty Acid (SCFA and BCFA) Quantification

SCFA/BCFA formation was assessed in frozen fecal slurries (1:4 dilutions) by gas chromatography (GC) following standard procedures [36]. The SCFA/BCFA concentrations (mM per g fecal sample) were calculated in duplicate from standard curves based on the internal standard 0.1 M 2-Ethyl butyric acid. An external standard (a mixture of volatile fatty acids and salts [SCFA: 30 mM Acetic acid, 20 mM Propionic acid, 20 mM Butyric acid, and 5 mM Valeric acid; BCFA: 5 mM iso-Valeric acid, and 5 mM Iso-Butyric acid; salts: 10 mM sodium formate; 10 mM Lithium lactate, and 10 mM sodium succinate]) was included in each GC run. In summary, peak sizes in samples were compared to the standards with respect to retention time and peak size during the GC run and output was subsequently validated manually and corrected for the dilution factor of the fecal slurry. Fecal pH was measured directly in the fecal slurries using a pH meter (WPA CD70 pH meter, Linton, Cambridgeshire, UK, with a Fisher brand sealed electrode FB68801).

### 2.5. DNA Extraction for Microbial Community Profiling

DNA was extracted from homogenized fecal samples using the FastDNA Spin kit for soil (MP Biomedicals, UK). The 450 μL homogenized samples were placed in lysing matrix E tubes and processed immediately or after freezing (−70 °C) for up to 2 weeks. Frozen samples were thawed on ice after adding 978 μL of sodium phosphate buffer and 122 μL MT buffer to each tube, with the same volumes added to the fresh samples, and processing continued following the manufacturer’s instructions. DNA was eluted in 100 μL FastPrep elution buffer and quantified by Qubit 2.0 Fluorometer (Life Technologies Ltd., Paisley, UK). Purified DNA was stored at −70 °C prior to bacterial community profiling.

### 2.6. PCR Amplification and 16S rRNA Amplicon Sequencing for Microbial Community Profiling

An amplicon library was generated by PCR amplification of the V1-V2 hypervariable region of the bacterial 16S rRNA genes using the barcoded primers MiSeq-27F, Eurofins Genomics UK Ltd., Wolverhampton, UK (5′-AATGATACGGCGACCACCGAGATCTACACTATGGTAATTCCAGMGTTYGATYMTGGCTCAG-3′) and MiSeq-338R (5′-CAAGCAGAAGACGGCATACGAGAT-barcode-AGTCAGTCAGAAGCTGCCTCCCGTAGGAGT-3′), which also contain adaptors for downstream sequencing using the Illumina MiSeq v3 system (Illumina Centre, Cambridge, UK). The reverse primers contained a unique (12 base) barcode specific to individual samples. Initial PCR amplification was performed with New England BioLabs Q5 High-fidelity DNA Polymerase (Catalogue number M0491L) (Hitchin, UK). Each individual reaction mix contained a DNA template (1 μL), 5× Q5 Buffer (5 μL), 10 mM dNTPs (0.5 μL), 10μM forward primer (1.25 μL), 10μM reverse primer (1.25 μL), Q5 Taq (0.25 μL), and sterile, deionized water (15.75 μL), giving a final volume of 25 μL. PCR cycling conditions were: 2 min at 98 °C; 20 cycles of 30 s at 98 °C; 30 s at 50 °C; 90 s at 72 °C; 5 min at 72 °C followed by a holding temperature at 10 °C. Four replicate PCR reactions were set up per DNA sample to ensure an adequate yield of amplicons. Following confirmation of amplification of appropriately sized products, the quadruplicate reactions were pooled and concentrated via NaCl and ethanol precipitation before being concentrated and quantified using a Qubit 2.0 Fluorometer (Life Technologies Ltd., Paisley, UK). A sequencing master mix was prepared using equimolar concentrations of DNA from each sample and was then cleaned up using AMPure XP magnetic beads (Beckman Coulter, High Wycombe, UK; Catalogue number A63880) following manufacturer’s instructions. Negative controls, in which water replaced both the homogenized fecal sample during DNA extraction and the DNA during PCR amplification, were prepared. These negative controls and a positive control of a defined DNA community mix (ZymoBIOMICS™ Microbial Community Standard, Cambridge Bioscience, Cambridge, UK) were also amplified with the MiSeq primers and were included in the sequencing runs to verify a lack of contamination and serve as quality control of the data. Sequencing was carried out on an Illumina MiSeq v3 flow cell producing 300 bp paired-end reads. Raw sequencing data has been deposited to the European Nucleotide Archive database under accession number PRJEB64619.

### 2.7. Dietary Analysis

Examples of standard weekly menus were obtained from nine participating care homes (accounting for 82% of participants). Mean daily macronutrient intake (g/day) was calculated using an electronic version of McCance and Widdowson’s ‘the composition of foods’ [37]; WinDiets software (Professional Version, Robert Gordon University, Aberdeen, UK, 2017).

### 2.8. Bioinformatics and Statistical Analysis

Sequenced reads were automatically demultiplexed within the Illumina MiSeq system using the inbuilt bcl2fastq tool. *DADA2* was used to process raw reads and identify amplicon sequence variants (ASVs) [38]. Primers were removed and reads were trimmed and quality filtered before applying the core *DADA2* algorithm for merging and chimera removal. This gave between 49,390 and 317,784 reads per sample, with an average of 201,906. Taxonomy was assigned to ASVs using the *DADA2* implementation of the naïve Bayesian classifier method and the SILVA database (version 138) [39]. There were 7506 unique ASVs identified, with 6125 carried forward in the analysis after eliminating singletons (ASVs present only once, in a single sample). For individuals with two samples, diversity metrics were calculated separately for each replicate and the average was used. For abundance plots and testing, the mean ASV counts for each individual were calculated and used. Rarefaction curves for each of the alpha diversity metrics plateau, illustrating that sample diversity was adequately covered with the sequencing depth obtained. Core diversity analyses were computed in *PhyloSeq* and plotted using *ggplot2*. Kruskal–Wallis and Wilcoxon rank sum tests were performed to compare three alpha diversity measures (observed species, Shannon Index, and Simpson Index) between groups. Bray–Curtis beta diversity measurements were used to assess dissimilarity of microbiota composition between samples. Statistical testing for changes in beta diversity in response to sample variables was performed using PERMANOVA tests implemented in R package vegan [40]. Differential abundance testing of taxa between groups was performed with corncob. For each sample, ASV counts were merged at set taxonomic levels (phylum, family, and genus) and a beta-binomial regression model was used to test for significant differences in abundance between sample groups (while including care home as a covariate). Taxa were considered differentially abundant where the false discovery rate (FDR) (Benjamini–Hochberg-adjusted *p* value) was <0.05. The relative abundances of the different taxa in the samples were visualized using *ggplot2*.

The sequence data from the positive control broadly matched the expected composition, although Salmonella enterica and Escherichia coli were overrepresented and Lactobacillus fermentum underrepresented. In contrast, the negative controls, containing water in place of a sample during both DNA extraction and PCR amplification, had only 15 and 16 sequence reads, respectively, all of which were removed during quality filtering, indicating that there was no contamination present in the samples.

Bacterial abundance and SCFA data were analyzed by linear mixed models in the R package lme4 to account for the variable number of samples (1 or 2) available for each volunteer. Random effects were volunteer ID and variation within volunteer, while fixed effects were age, gender, and group. As many variables showed a skewed distribution, we also performed this analysis on log-transformed variables, and whenever the Shapiro–Wilk test statistic was <0.9), *p*-values were reported from the log scale analysis if the corresponding SW > 0.9. Principal component analysis of the SCFA and bacterial abundance data, based on the correlation matrix, was carried out to look for overall patterns in the differences between the groups. Data was also compared with a cohort of healthy adult values (combined average values from up to 158 free-living healthy individuals collected in ten separate human studies [41]).

Data were analyzed using SPSS (IBM SPSS Statistics version 28.0.0.0) and R 4.2.2 (R Foundation for Statistical Computing, Vienna, Austria). Correlation analysis was performed in SPSS to calculate the Spearman rank-order correlation coefficient. This non-parametric measure of correlation was chosen as the data were not normally distributed. Values for Spearman rank-order correlation coefficients were calculated for each pair of variables, ranging from −1 (perfect negative [inverse] correlation) to +1 (perfect positive correlation). Correlations were calculated for all participants who had data for all variables (*n* = 43) to identify any differences between the control and AD groups. Correlation coefficients denote >±0.4 for a moderately strong, >±0.6 for strong, and >±0.8 for a very strong correlation.

The analysis scripts and source data are available through the following Figshare link [https://dx.doi.org/10.6084/m9.figshare.25151639] as well as through the DOI—10.6084/m9.figshare.25151639.

## 3. Results

### 3.1. Study Group Participants

From the 46 care homes contacted, only 16 were willing to participate in the study. Unfortunately, not all recruited participants provided samples, and additionally, we did not receive duplicate samples from all participants. The final numbers of participants providing at least one sample were: C (*n* = 19) and AD (*n* = 24), with the AD group further divided into AD (*n* = 14) and ADCB (*n* = 10). The characteristics of the participants are shown in (Table 1).

### 3.2. Short-Chain and Branched-Chain Fatty Acid (SCFA and BCFA) Profiles

Fecal pH ranged between 7 and 7.6, though the mean pH from controls tended to be lower (7.1 ± 0.76) than samples obtained from the AD cohort (7.4 ± 0.58). There also seemed to be an effect of gender on fecal pH, with the average pH in females slightly higher than males (7.38 vs. 7.11; *p* = 0.04), although this could be affected by the higher numbers of females recruited (15 males versus 28 females).

**Figure 2:**
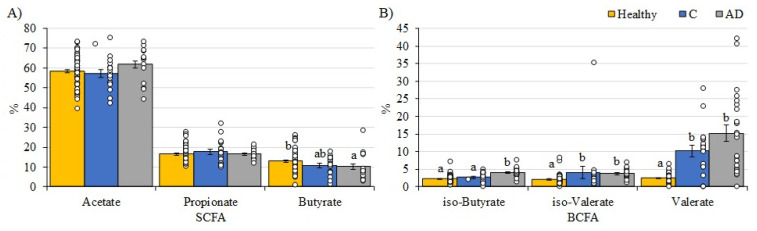
Percentages of specific fatty acids as proportions of total fatty acids detected in fecal samples from the AGE-GB study cohorts (Controls—Blue; AD—Grey) compared to a healthy adult (yellow). (**A**) SCFA; (**B**) BCFA. Healthy adults are values from 78 free-living healthy individuals [41]. Mean values (+/− standard error of the mean) and individual values are plotted. Superscripts indicate significant differences (*p* < 0.05) between values. Note the *y*-axis scale is different between panels (**A**,**B**).

The SCFA profiles between replicate samples 1 and 2 from participants providing two samples were relatively consistent, indicating that there is temporal stability in metabolite production by the gut microbiota in individuals residing in a care home environment. The care home residents with AD tended to have lower total concentrations of measured fatty acid metabolites than the free-living healthy adult cohort [41]. The mean total SCFA concentration was higher in healthy adults (84.8 mM) and the AGE-GB control cohort (85.7 mM) than in the combined AD group (70.4 mM). This reflected the lower concentrations of each of the three major SCFA, although the high level of inter-individual variation meant this was not statistically significant. Proportionally, there was a higher percentage of acetate in the AD group compared to the control group (*p* = 0.065) and the healthy adults (*p* = 0.072), and a lower percentage of butyrate in both the AGE-GB control and AD groups compared to healthy adults (*p* = 0.143 and *p* = 0.033, respectively, Figure 2). The most significant differences were in the concentrations of branched-chain fatty acids (BCFA) with considerably more BCFA as a proportion of total SCFA in each AGE-GB cohort compared to healthy adults (*p* = 0.026, AGE-GB control; *p* < 0.001, AD). The percentages of iso-butyrate were similar in healthy adults and the control cohort and were significantly higher in the AD group (*p* < 0.001), while percentages of iso-valerate (*p* = 0.014) and valerate (*p* < 0.001) were both significantly lower in the unlinked group of free-living healthy adults than any care home residents (Figure 2).

### 3.3. Microbiome Analysis

Comparison of the microbial composition between the different cohorts identified some clear differences, although the high level of inter-individual variation made it difficult to identify group-specific trends.

**Figure 3:**
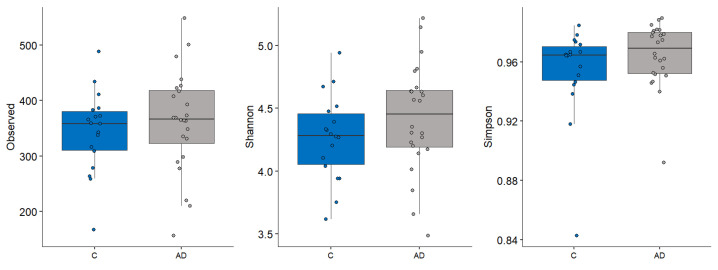
Different alpha diversity metrics showing no significant difference in the microbial diveryes tsity between control (blue) and those with AD (grey).

**Figure 4:**
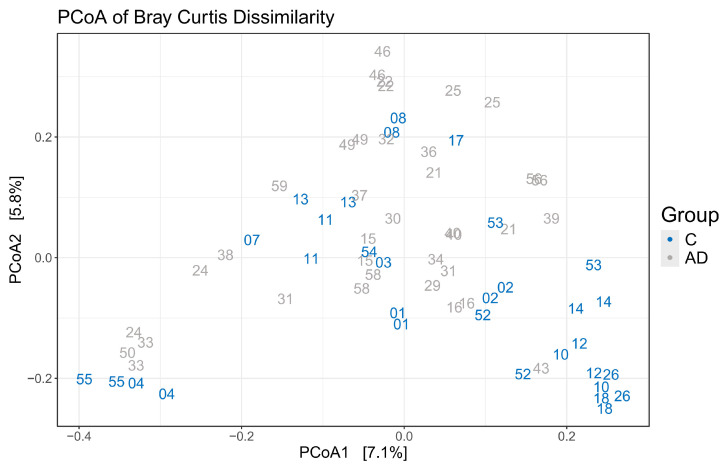
Principle coordinate analysis to illustrate grouping of samples based on Bray–Curtis dissimilarity showing replicate samples from the same volunteer (same numbers) clustered closely together.

Alpha diversity metrics, measuring the within group diversity, found no significant differences in microbial diversity between the control and AD cohort (Figure 3). Bray–Curtis dissimilarity calculations showed that the two replicate samples from the same volunteer (when available) clustered closely together (Figure 4), justifying the use of the mean scores for group comparisons. PERMANOVA testing of volunteers was confirmed to be a significant factor affecting microbial composition, with volunteer differences explaining 90.7% of the variation in sample composition in the unmerged dataset (*p* = 0.0001). Additional PERMANOVA testing of the Bray–Curtis dissimilarity matrix showed that overall, the microbial composition was influenced by care home (R2 = 0.30668, *p* = 0.042) but not cohort (R2 = 0.02587, *p* = 0.2133). The effect of care home is likely due to the uneven distribution of participants residing in different care homes among the study groups. For instance, 4/13 care homes recruited only individuals to the control group. This variable was thus included in models testing other variables. Age and gender had no significant impact on microbial composition. There were also no significant compositional differences between the two AD groups (AD versus ADCB) when they were compared directly (*p* = 0.083).

**Figure 5:**
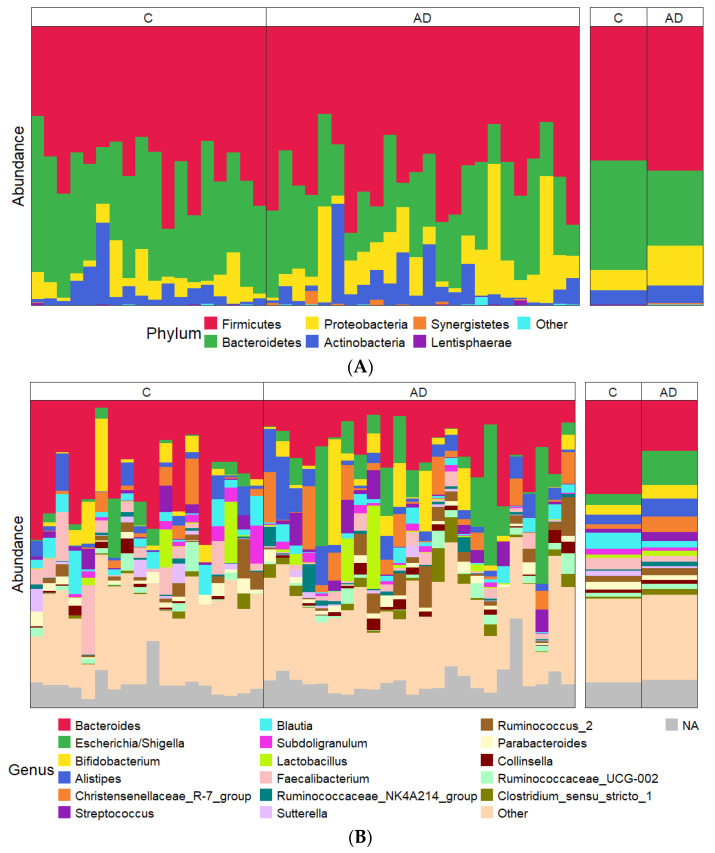
(**A**,**B**) Relative abundance of most abundant taxa showing data for each individual sample on the left and combined study groups C and AD on the right. (**A**) Phylum level; (**B**) genus level. The most abundant 17 genera across the entire sample set are shown in order of average abundance (top to bottom).

Taxonomic profiling at the phylum level showed a lower abundance of Bacteroidota (Bacteroidetes) and an increased abundance of Pseudomonadota (Proteobacteria) in the AD groups compared to the controls (Figure 5A), although neither of these changes were statistically significant. The large degree of inter-individual variation observed became even more apparent when comparing samples at a genus level (Figure 5B).

**Figure 6:**
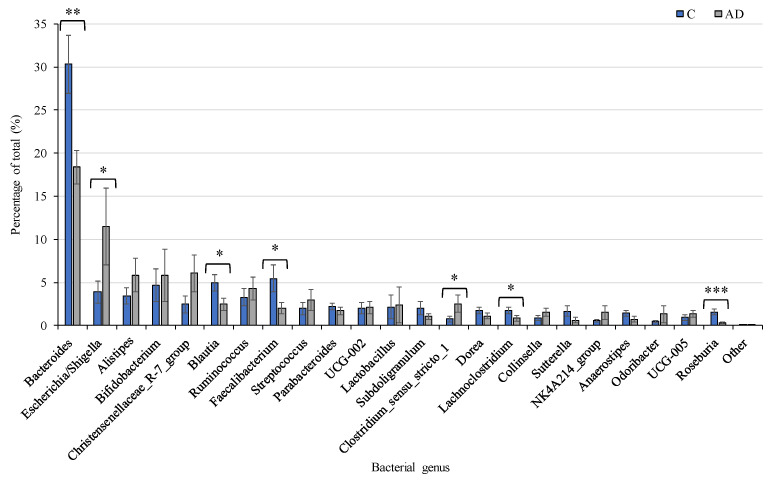
Relative abundance of the 23 most prevalent bacterial genera present at >1% of total in Controls (*n* = 19, Blue), compared to the combined AD groups (*n* = 24, Grey). Mean values (+/− standard error of the mean) are plotted. Significant differences between groups are indicated by asterisks (*, **, *** correspond to *p* < 0.05, *p* < 0.01, *p* < 0.001 respectively).

Despite the overall inter-individual variation, significant differences were found in the abundance of specific taxa between the study groups, with marked differences between the control group and the combined AD groups. Focusing analysis on the top 23 genera present at more than 1% abundance (Figure 6), several bacterial taxa were identified whose percentage abundance differed significantly between groups. Levels of *Escherichia*/*Shigella* (*p* = 0.04) and Clostridium_sensu_stricto_1 (*p* = 0.03) were both significantly higher in the AD group compared to the controls. In contrast, the control group had significantly higher levels of *Bacteroides, Blautia, Roseburia*, and *Faecalibacterium* genera (*p* = 0.002, *p* = 0.02, *p* = 0.0001, *p* = 0.02 respectively) than the AD group. Average counts of the genus *Collinsella* varied but tended to be higher in AD (1.5%) than control (0.9%) cohorts, although this did not reach significant levels (Figure 6). Additionally, there were very few differences observed between the separated AD and ADCB groups (Supplementary Figure S1).

Due to the apparent contrast in relative abundance of bacteria generally associated with health compared to those linked with inflammation, we performed a combined analysis revealing an even greater difference between the cohorts. Combined numbers of the pro-inflammatory species *Collinsella*, *Clostridium_sensu-stricto 1*, and *E. coli*/*Shigella* were significantly higher in the AD (average 16.18%) compared to the control (6.11%) cohort (*p* = 0.007). At the same time, the combined abundance of beneficial bacteria (*Bacteroides, Blautia, Roseburia* and *Faecalibacterium* species) were significantly higher in the control (41.8%) than the AD (22.3%) cohorts (*p* = 0.0001) (Supplementary Figure S2).

### 3.4. Assessment of Gut Inflammation

Concentrations of calprotectin, an inflammatory biomarker found in feces, were used as a proxy to assess levels of possible intestinal inflammation. Average calprotectin concentrations showed considerable inter-individual variation between all volunteers. Subsequent one-way ANOVA testing revealed no significant differences between the healthy and AD groups, although there was a tendency for the AD group to have slightly higher calprotectin levels (Supplementary Figure S3). All levels detected were lower than the 50 mg/kg concentration indicative of intestinal inflammation.

### 3.5. Diet and the Gut Microbiota

It was not possible to link microbial changes to specific dietary intake at an individual level as there was no information about individual food selection from the menus provided by the care homes. Based on the menus, the average daily nutritional value of the food offered in each of the care homes was similar and in line with the applicable guidelines (Supplementary Table S2). Notably, dietary estimates for daily protein intake were higher than adult recommendations, which were consistent with the requirement for more protein consumption in older adults to preserve muscle mass. In contrast, estimates of daily fiber intake were lower (11–18 g/day) than the 30 g/day adult recommendations.

### 3.6. Correlations Between Data Sets

**Figure 7:**
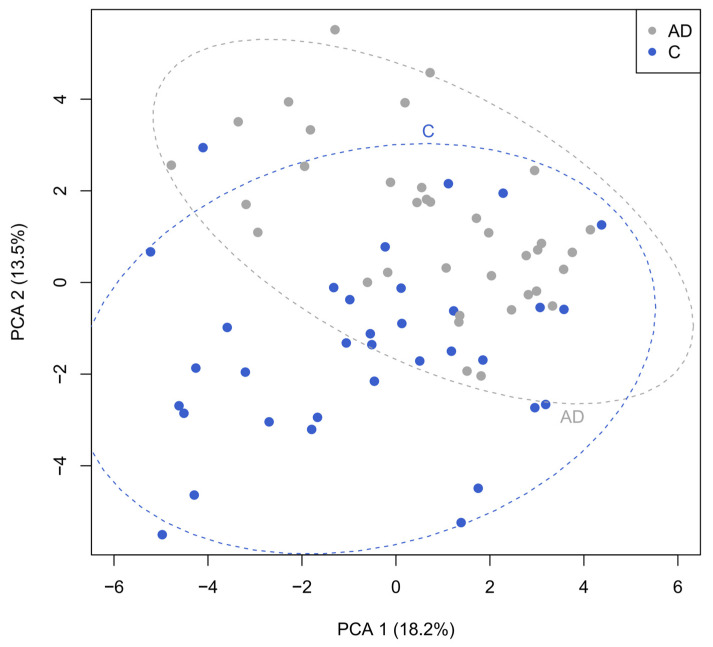
Principal component analysis investigating correlations between bacteria and fecal metabolites revealing the clear separation between the control (blue) and AD cohorts (grey).

Correlation analysis was performed to determine any interesting relationships between the different datasets, both for the full population and the differences between the control and AD participants. Principal component analysis focused on bacteria and fatty acid metabolites showed a clear separation between the control group and individuals in the AD groups (Figure 7). There was a positive correlation between concentrations of specific short-chain fatty acids (percentages detected in fecal samples) and percentage abundance of specific bacterial groups in the whole dataset (Supplementary Figure S4). For instance, butyrate concentrations positively correlated with relative abundance of *Faecalibacterium*, *Lachnoclostridium*, *Blautia*, and *Roseburia* species, and there were differences between controls and individuals with AD (Supplementary Figure S5). Concentrations of calprotectin positively correlated with prevalence of the grouped pro-inflammatory bacteria and negatively correlated with the abundance of beneficial bacterial groups (Supplementary Figures S4 and S5).

## 4. Discussion

These results indicate clear differences in the relative abundance of certain bacterial species and bacterial metabolites between care home residents with and without Alzheimer’s disease that could be indicative of variable gut-brain axis activity. The AD cohort had significantly higher proportions of pro-inflammatory bacterial species and fewer ‘beneficial bacteria’. We also found clear correlations between concentrations of beneficial bacterial metabolites and abundance of ‘healthy bacteria’. While numerous studies have investigated the role of the gut-brain axis in AD, to the best of our knowledge this is the first study to both analyze the microbial function and to attempt to compare the gut microbiota composition in participants with AD and those with AD plus BPSD (the subgrouping ADCB). In line with other studies, we identified an altered AD gut microbiome compared to controls. Unfortunately, we were unable to observe any clear differences between the two AD groups, either because none exist or because the sample sizes were too small when the AD cohort was separated into the two subgroups.

AD victims had increased levels of *Escherichia/Shigella* and *Clostridium_sensu_stricto_1*, which are linked to higher levels of gut inflammation. *Escherichia/Shigella* species can lead to higher levels of circulating lipopolysaccharide (LPS) [42] and have been found in greater concentrations in the gut microbiota of individuals with mild cognitive impairment and in several prior AD studies [22,23,43]. Certain strains of *Escherichia/Shigella* are known to form amyloid protein structures, known as curli, similar to those seen aggregating in the brains of AD victims [43,44]. Although this is not definitively linked, it does raise one possibility as to how high levels of *Escherichia/Shigella* could potentially contribute to increased Alzheimer’s pathogenicity [44]. A positive correlation between the abundance of the pro-inflammatory genus *Collinsella* and the AD phenotype has also been observed [45]. Numbers of *Collinsella* sp. were higher in AD than control cohorts in our study, although this did not reach significant levels. However, when numbers of three known pro-inflammatory taxa were combined, there were significantly more in the AD group than the control cohort. At the same time, the numbers of bacteria generally classified as being beneficial for health were depleted in the AD cohort compared to the control. Due to the small number of individuals in each separated AD cohort (AD, *n* = 14; ADCB, *n* = 10), the minor differences between them (such as the slightly higher number of pro-inflammatory bacteria and the slightly higher alpha diversity in ADCB) were not significant.

Similar to other studies, the AD cohort had decreased relative abundance of *Bacteroides*, *Faecalibacterium*, *Blautia*, and *Roseburia* species which are typically linked with good health. Both *Roseburia* and *Faecalibacterium* sp. are key butyrate producers and a significant decrease in the number of butyrate-producing bacteria, and subsequently butyrate, has previously been associated with AD [46]. What cannot be determined from our data is whether the difference in microbiota is contributing to AD pathogenicity or whether AD itself causes the microbial dysbiosis.

The specific alterations in gut microbiota in those with AD, identified through differences in the taxonomic composition of fecal samples, differs between published studies [22]. One study found that individuals with AD had decreased microbial diversity and a reduced abundance of Firmicutes and *Bifidobacterium*, and increased Bacteroidetes [47], while a study from China also showed a reduced abundance of Bacteroidetes and a slightly higher abundance of Actinobacteria among individuals with AD [48]. Differences between studies may be explained by dietary differences between countries, but it is important to note that in all cases, groups of controls could be differentiated from those with AD by their gut microbiota profile. It will be interesting to see whether additional studies with larger datasets, such as the MiBioGen consortium, can provide more insight into potential interactions between host factors and abundance of specific bacterial genera and different types of AD [45].

One way through which alterations in the gut microbiota could influence the impaired cognitive function associated with AD is via changes in bacterial metabolite production. Microbial metabolites produced in the gut are recognized factors involved in gut–brain signaling, as reviewed in Parker et al. (2019) [49]. The mean total concentration of SCFA was lower in the AD cohort than both the control and younger healthy adult cohorts, and participants with AD had significantly increased proportions of the BCFA iso-butyrate. SCFAs are predominantly produced by carbohydrate fermentation, and branched chain fatty acids (BCFAs) are the products of protein metabolism and are produced in smaller amounts [50]. Animal studies using AD transgenic mice also found significantly reduced SCFA levels [51], in addition to other human studies of the dysregulated gut [21]. Separate experiments with AD mice indicated that supplementation with a mixed species probiotic (a mix of nine bacterial strains) led to increased fecal concentrations of SCFA, while concentrations of circulating pro-inflammatory cytokines decreased [52]. Feeding mice a high fiber diet increased numbers of butyrate-producing bacteria and butyrate concentrations, and improved cognition [53], which is one of the numerous health benefits of the ‘Mediterranean diet’ (reviewed in [54]). Importantly, selected SCFA products of dietary fiber fermentation (particularly valerate, butyrate, and propionate) have also been shown in vitro to interfere with the assembly of β-amyloid peptides into neurotoxic aggregates [55]. It is postulated that SCFAs have an important role in maintaining the integrity of the blood–brain barrier and protection from oxidative stress [56]. Less is known about BCFAs and their influence on brain health, but proportions of BCFA have been shown to change with aging and with dietary fiber intake [57]. It is important to note that concentrations of SCFAs measured in feces are a balance of production, utilization, and absorption and may not always reflect the amount of SCFA circulating. Indeed, in a study where butyrate was delivered directly to the colon in capsules, there was no change in fecal concentrations, although concentrations of all three main SCFAs were elevated in blood serum [58].

Specific bacterial metabolites and bacterial genera/species also have an impact on gut inflammation. The significantly higher numbers of pro-inflammatory bacteria and the heightened fecal concentration of iso-butyrate in the AD cohort compared to the control cohort correlated with slightly higher concentrations of calprotectin. High levels of calprotectin (mean 140 mg/kg) have previously been observed in individuals with AD [59]. None of the individuals in our study had the elevated levels of calprotectin associated with gut inflammation. The difference in our results and those found by others may relate to the stage of the disease, but it is possible that the trend of increased calprotectin will become clearer with a larger sample size.

We hoped to match gut microbiota composition and function with dietary intake in the study. Unfortunately, the reliance on care home staff to complete dietary records for participants with AD meant this was not feasible given the demands on staff time. Analysis of the menus proved only that dietary recommendations were followed, and the ability for individuals to choose from the menus meant these alone were insufficient in making any conclusions. The menus do suggest that while protein provision seems appropriate in preventing sarcopenia in older people, dietary fiber content was low. As indicated above, this could impact microbiota composition and metabolite production and may be an area for further investigation.

A real limitation of this study was the small cohort size and the difficulties in obtaining fecal samples from enough participants to separate the AD group into AD and ADCB cohorts. This reflects some of the challenges of conducting research within the care home environment where staff are stretched thin and where multiple people may be involved in the care of an individual participant. This particular study was dependent on the care home staff being trained in supporting participants with sample collection. In order to avoid the study being unduly arduous for both care home staff and participants we were also unable to collect additional data on individual physical activity, although this is unlikely to vary greatly between different care home residents. Despite these issues, it is important that care home residents are represented in research studies, and that support is available to conduct future research studies within this environment [60,61].

One important finding from this study was the reproducibility of results from samples obtained one to two weeks apart from a single individual. Although less than half the participants (47%) provided the requested two samples, and despite the high level of inter-individual variation, repeat samples from the same participant showed a high level of clustering in both the SCFA and microbiota analysis, suggesting the composition of these elderly individuals’ gut microbiota was fairly stable. Similarly, the Eldermet Study reported that older adults had a high degree of inter-individual variability of their gut microbiota profiles but showed temporal stability over a period of several months [20]. Thus, future studies comparing single fecal samples from this demographic of participants could be considered adequate, significantly reducing the burden of the study on participants, care home staff, and researchers.

## 5. Conclusions

Overall, this study supports the growing body of evidence for the role of an altered gut microbiota in the pathogenesis of AD, illustrating that changes in both microbial composition and metabolite production are important. Establishing whether this has any role in the altered behaviors noted in AD, along with additional challenging behaviors, requires further research. This should include studies into whether maintenance of a healthy gut microbiota can delay the onset of AD, and whether influencing the gut microbiota through diet, prebiotics, probiotics, or direct microbial transplantation can reduce AD symptoms.

## Figures and Tables

**Table 1:** Participant characteristics and the number of fecal samples collected (*n* = 43).

Group	Sex		Mean Age ± SEM (Range)	First Sample Collected	Second Sample Collected
	Male (*n* = 15)	Female (*n* = 28)			
C (*n* = 19)	7	12	86.1 ± 2.24 (67–97)	19	15
AD (*n* = 24)	8	16	85.9 ± 1.48 (67–97)	24	13

AD—Participants with Alzheimer’s Dementia; C—Control participants. Note that the AD group was further split into AD (*n* = 14) and ADCB (*n* = 10) for some analyses. The characteristics of the divided group are shown in Supplementary Table S1.

## Data Availability

The datasets generated for this study can be found in the European Nucleotide Archive, accession number PRJEB64619. These data are private until acceptance of the manuscript.

## Supplementary Materials

The following supporting information can be downloaded at: https://www.mdpi.com/article/10.3390/geriatrics10020037/s1, Supplementary Figure S1: Relative abundance of the 23 most abundant bacterial genera present at >1% of total in Controls (*n* = 19, Blue), those with AD (*n* = 14, Grey) and those with ADCB (*n* = 10, Orange). Mean values (+/− standard error of the mean) are plotted; Supplementary Figure S2: Bar chart showing average relative abundance of pro-inflammatory bacteria; Supplementary Figure S3: Bar chart showing average Calprotectin levels by group (mg/kg) +/− S.E; Supplementary Figure S4: Heatmap of correlations showing the relationship between the relative abundances (%) of the six main SCFA/BCFA metabolites and the calprotectin concentration with the most abundant 23 bacterial taxa; Supplementary Figure S5: Scatterplots illustrating associations between four bacterial genera and butyrate concentrations (panels A to D). The associations between pro-inflammatory/beneficial bacterial groups and calprotectin concentrations are shown in the scatterplots in panels E and F; Supplementary Table S1: Participant characteristics; Supplementary Table S2: Mean daily nutritional composition of foods provided by care homes based on menu analysis; Supplementary Methods [62].
