# Supplementary material for: Aging Gut-Brain Interactions: Pro-Inflammatory Gut Bacteria Are Elevated in Fecal Samples from Individuals Living with Alzheimer’s Dementia
Source: Geriatrics (Basel). 2025 Mar 7;10(2):37. doi: 10.3390/geriatrics10020037 (PMC11932241; doi:10.3390/geriatrics10020037)
Supplement: Supplementary file 1 [file geriatrics-10-00037-s001.zip › geriatrics-3372472-SI.pdf]

Supplementary Material

1 Supplementary Data

1.1 Supplementary Figures

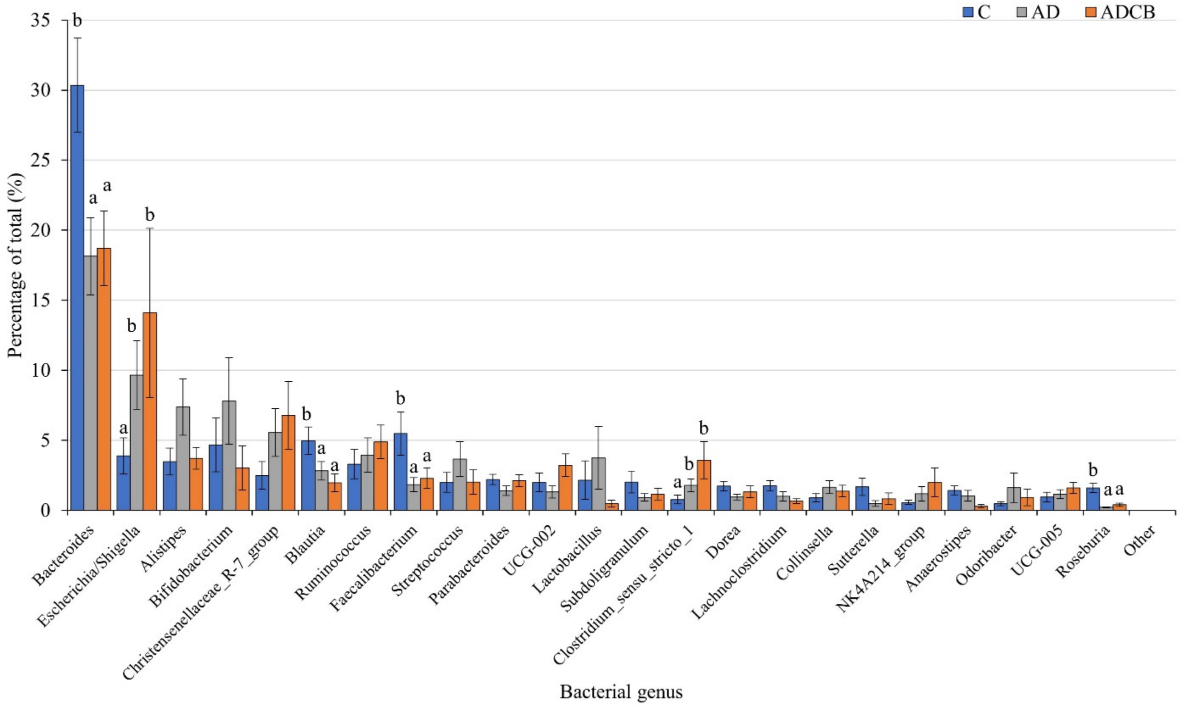

Supplementary Figure S1. Relative abundance of the 23 most abundant bacterial genera present at >1% of total in Controls (n=19, Blue), those with AD (n=14, Grey) and those with ADCB (n=10, Orange). Mean values (+/- standard error of the mean) are plotted. Different superscripts indicate significant differences ($p < 0.05$) between values.

AD, Participants with Alzheimer's Dementia; ADCB, Participants with Alzheimer's Dementia and Challenging Behavior associated with BPSD (BPSD, Behavioral or Psychological Symptoms of Dementia); C, Control participants.

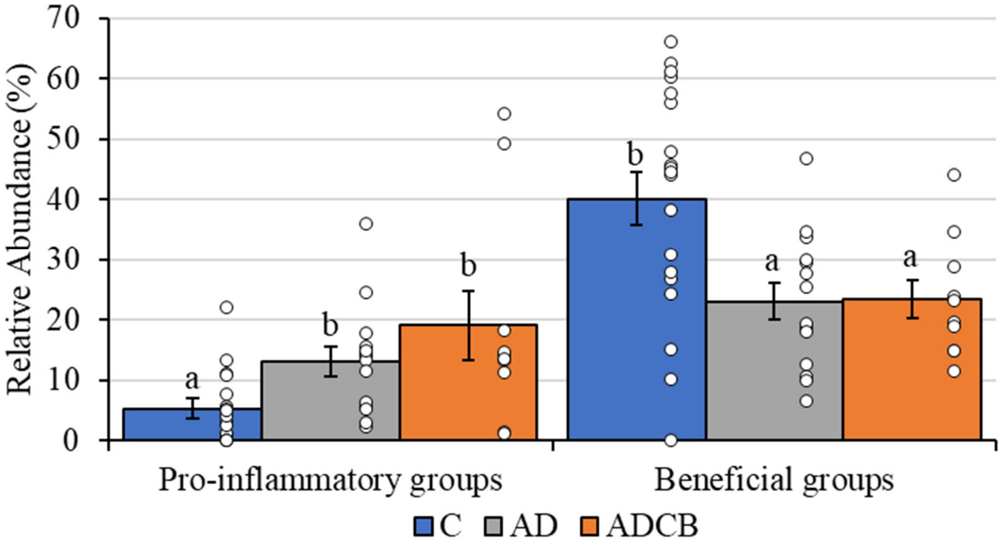

Supplementary Figure S2. Bar chart showing average relative abundance of pro-inflammatory bacteria (classed as *Collinsella, Clostridium_sensu-stricto* 1 and *E. coli/Shigella* species) and beneficial bacteria (*Bacteroides*, *Blautia*, *Roseburia* and *Faecalibacterium* species) in the three cohorts, +/- standard error of the mean.

For additional information, the two AD subgroups (AD -grey; and ADCB - orange) are shown in different colors compared to controls (blue).

AD, Participants with Alzheimer's Dementia; ADCB, Participants with Alzheimer's Dementia and Challenging Behavior associated with BPSD (BPSD, Behavioral or Psychological Symptoms of Dementia); C, Control participants.

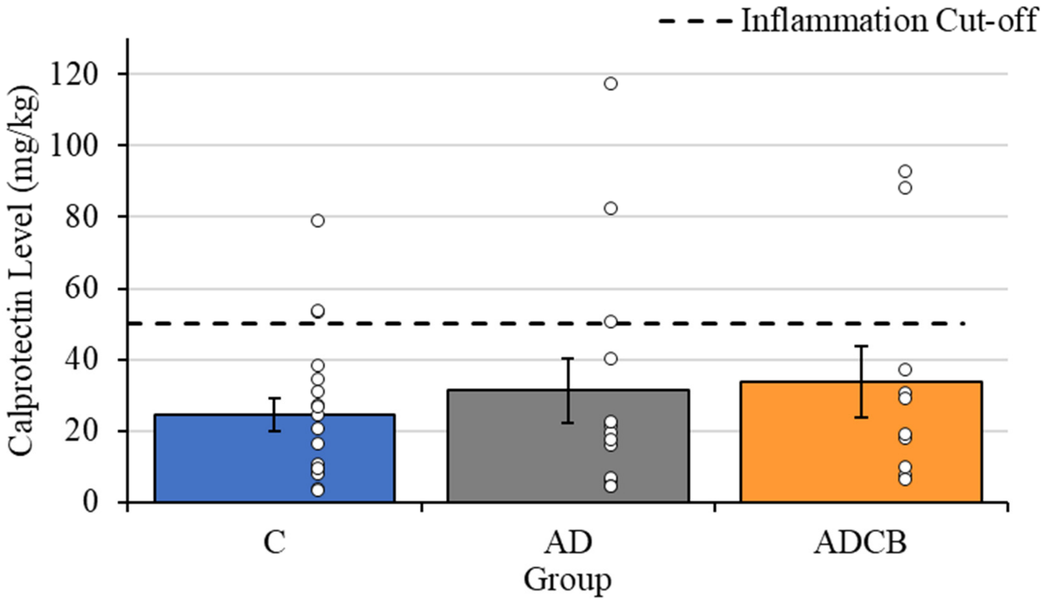

Supplementary Figure S3. Bar chart showing average Calprotectin levels by group (mg/kg) +/- S.E.
A value of 50mg/kg is the cut-off above which an individual is considered to have gut inflammation.
For additional information, the two AD subgroups (AD -grey; and ADCB - orange) are shown in different colors compared to controls (blue).

AD, Participants with Alzheimer's Dementia; ADCB, Participants with Alzheimer's Dementia and Challenging Behavior associated with BPSD (BPSD, Behavioral or Psychological Symptoms of Dementia); C, Control participants.

	Acetate %	Propionate %	iso-Butyrate %	Butyrate %	iso-Valerate %	Valerate %	Calprotectin
Bacteroides %	**-0.42**	0.23	**-0.44**	0.17	0.08	-0.24	**-0.34**
Escherichia/Shigella %	-0.02	-0.12	0.22	0.21	-0.05	**0.32**	**0.26**
Alistipes %	**0.27**	0.08	-0.11	-0.18	-0.07	-0.11	-0.05
Bifidobacterium %	**0.28**	-0.07	0.07	**-0.32**	-0.05	-0.18	-0.10
Christensenellaceae_R-7_group %	**0.49**	**-0.25**	**0.29**	**-0.52**	0.11	-0.22	-0.02
Blautia %	-0.18	0.13	**-0.27**	**0.27**	**-0.25**	-0.02	-0.23
Ruminococcus %	0.05	-0.14	0.21	0.00	0.14	0.23	-0.06
Faecalibacterium %	-0.19	-0.07	-0.17	**0.25**	0.00	0.12	-0.09
Streptococcus %	-0.04	-0.16	0.11	0.16	-0.06	0.23	**0.44**
Parabacteroides %	-0.11	**0.34**	-0.22	0.01	-0.16	-0.18	-0.13
UCG-002 %	0.02	-0.24	**0.31**	-0.11	0.20	0.04	0.22
Lactobacillus %	0.02	0.16	0.02	-0.07	-0.04	-0.02	**0.44**
Subdoligranulum %	0.23	-0.20	-0.15	-0.01	-0.02	0.04	-0.05
Clostridium_sensu_stricto_1 %	-0.07	-0.11	**0.29**	0.09	0.18	**0.25**	0.12
Dorea %	-0.06	0.17	-0.14	**0.25**	-0.17	-0.11	**-0.25**
Lachnoclostridium %	**-0.29**	0.18	-0.15	**0.25**	-0.04	-0.12	**-0.29**
Collinsella %	-0.12	-0.18	**0.40**	0.14	0.04	0.22	-0.14
Sutterella %	**-0.32**	0.02	**-0.32**	-0.10	0.44	-0.07	-0.05
NK4A214_group %	**0.36**	-0.09	0.07	**-0.44**	0.09	-0.24	-0.17
Anaerostipes %	**0.25**	-0.03	-0.14	-0.24	-0.18	-0.19	**-0.26**
Odoribacter %	0.17	0.08	-0.17	-0.10	-0.06	-0.11	-0.06
UCG-005 %	0.22	-0.18	**0.29**	-0.19	0.04	0.02	0.04
Roseburia %	-0.07	0.03	**-0.46**	0.20	-0.19	-0.17	-0.18
Pro-inflammatory species	-0.05	-0.16	**0.32**	0.24	-0.01	**0.39**	**0.26**
Beneficial bacteria	**-0.43**	0.20	**-0.49**	**0.27**	0.00	-0.19	**-0.36**

Supplementary Figure S4. Heatmap of correlations showing the relationship between the relative abundances (%) of the six main SCFA/BCFA metabolites and the calprotectin concentration with the most abundant 23 bacterial taxa. Correlations between the groups of pro-inflammatory and beneficial bacterial species are also shown. Correlation values of <-0.25 (blue) or >0.25 (red) are statistically significant ($p < 0.05$) and are indicated in bold.

Amplicon Sequence Variants (ASVs) classified as pro-inflammatory bacteria - *Collinsella, Clostridium_sensu-stricto* 1 and *E. coli/Shigella.*

ASVs classified as beneficial bacteria - *Bacteroides, Blautia, Roseburia* and *Faecalibacterium.*

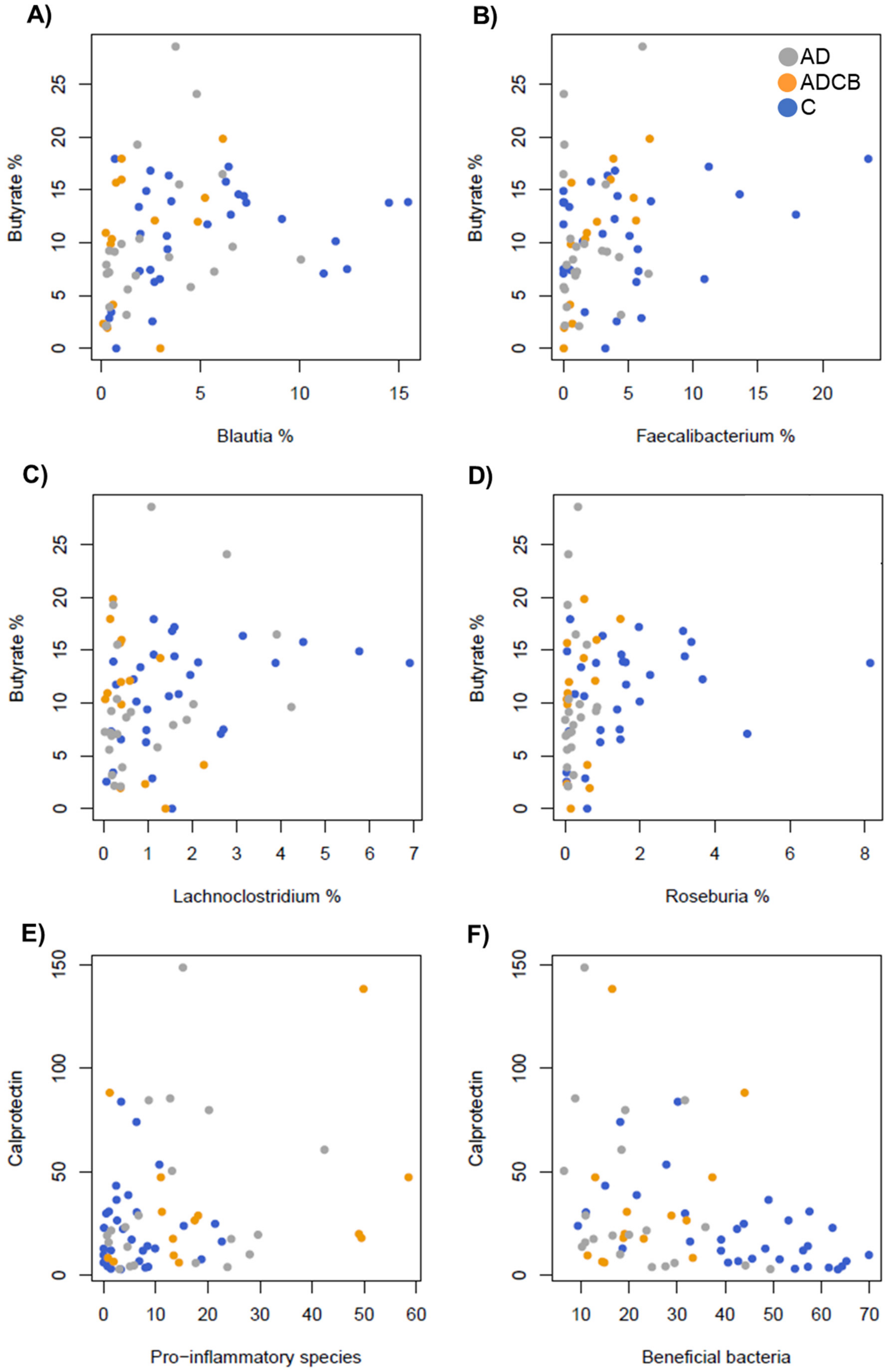
A)
B)
AD
ADCB
C
Butyrate %
Blautia %
Faecalibacterium %
C)
D)
Lachnoclostridium %
Roseburia %
E)
F)
Calprotectin
Pro−inflammatory species
Beneficial bacteria

Supplementary Figure S5. Scatterplots illustrating associations between four bacterial genera and butyrate concentrations (panels A to D). r values for specific correlations are shown in Figure S3.

The associations between pro-inflammatory/beneficial bacterial groups and calprotectin concentrations are shown in the scatterplots in panels E and F. r values for specific correlations are shown in Figure S3.

Amplicon Sequence Variants (ASVs) classified as pro-inflammatory bacteria - *Collinsella, Clostridium_sensu-stricto 1* and *E. coli/Shigella.*

ASVs classified as beneficial bacteria - *Bacteroides, Blautia, Roseburia* and *Faecalibacterium.*

For additional information, the two AD subgroups (AD -grey; and ADCB - orange) are shown in different colors compared to controls (blue).

1.2 Supplementary Tables

Supplementary Table S1. Participant characteristics (including separation into those with AD and those with AD+BPSD)

Group	Sex		Mean Age ± SEM (Range)	First sample collected	Second sample collected
	Male (n = 8)	Female (n = 16)			
AD (n=14)	4	10	86.0 ± 1.90 (74 - 97)	14	9
BPSD (n=10)	4	6	85.7 ± 2.50 (67 - 97)	10	4
AD + BPSD (n=24)	8	16	85.9 ± 1.48 (67 - 97)	24	13

Supplementary Table S2. Mean daily nutritional composition of foods provided by care homes based on menu analysis

Care Home	# weeks for which daily menus provided	*Mean daily macronutrient amounts (g/day)*			
		Fat	Protein	Carbohydrate	Fibre
A	6	113	80.5	287	16.0
B	4	89.9	91.1	273	12.0
E	4	88	86.4	238	13.6
H	3	109	97.4	320	17.5
I	8	113	93.8	292	17.1
J	4	100	88.4	270	14.2
K	4	102	95.2	277	16.1
M	4	91.6	94.1	267	10.7
N	12	113	98.3	325	15.7
Mean	5.44	102	91.7	283	14.8
SD	2.88	10.4	5.72	27.1	2.33

1.3 Supplementary methods: Participant Selection and Enrolment

1.3.1 Identifying participants

A research nurse approached local care homes to determine whether they were willing to be involved in the study. If interested, the care home manager was asked to identify individuals within the care home that potentially fell into each of the three planned study groups and ask if they would be willing to talk to the research team. On a subsequent visit the potential participants were approached and given information about the study, and asked if they would like to participate. Where an individual did not have capacity to consent themselves, their welfare attorney or nearest relative was approached to check if participation would be in line with their wishes. If there was any doubt about an individual's wish to participate they were excluded from the study.

1.3.2 Consenting participants

All potential participants were screened for capacity to consent to study involvement by the research team using a set proforma which included assessment using the 4 'A's test (4AT). The 4AT is a delirium screening tool that can also be used to pick up pre-existing cognitive impairment [62]. In assessing participant capacity, consideration was given to participant understanding of study information, ability of the participant to weigh up the pros and cons of involvement and ability of participant to retain information. Simplified participant information leaflets were made available to aid understanding. Where capacity was impaired or a Section 47 certificate of incapacity was already in place, consent was taken by proxy from the participant's welfare attorney/guardian or next of kin by an appropriately trained member of the research team.

1.3.3 Screening for eligibility

In order to be recruited into the Alzheimer's dementia study groups there had to be an existing medical diagnosis of Alzheimer's dementia and this was confirmed from care home records. Where the diagnosis of dementia subtype was unclear, this was clarified by contact with the participant's General Practitioner who were able to check the participant's primary care medical records. For recruitment to the healthy control group, records were checked to ensure there was no dementia diagnosis and no ongoing assessment for this. The sub-group stratification of Alzheimer's dementia patients with or without behaviours that are challenging (the ADCB cohort) was assigned after speaking to care home staff and analysing care home records of participants. Potential participants were also excluded if they had used antibiotics in the preceding eight weeks, or if they had known gastrointestinal disease.
